# Both identity and non-identity face perception tasks predict developmental prosopagnosia and face recognition ability

**DOI:** 10.1038/s41598-024-57176-x

**Published:** 2024-03-19

**Authors:** Rachel J. Bennetts, Nicola J. Gregory, Sarah Bate

**Affiliations:** 1 Division of Psychology, College of Health, Medicine and Life Sciences, Brunel University London, Kingston Lane, Uxbridge, UB8 3PH UK; 2 Department of Psychology, Bournemouth University, Poole, UK

**Keywords:** Human behaviour, Neurological disorders

## Abstract

Developmental prosopagnosia (DP) is characterised by deficits in face identification. However, there is debate about whether these deficits are primarily perceptual, and whether they extend to other face processing tasks (e.g., identifying emotion, age, and gender; detecting faces in scenes). In this study, 30 participants with DP and 75 controls completed a battery of eight tasks assessing four domains of face perception (identity; emotion; age and gender; face detection). The DP group performed worse than the control group on both identity perception tasks, and one task from each other domain. Both identity perception tests uniquely predicted DP/control group membership, and performance on two measures of face memory. These findings suggest that deficits in DP may arise from issues with face perception. Some non-identity tasks also predicted DP/control group membership and face memory, even when face identity perception was accounted for. Gender perception and speed of face detection consistently predicted unique variance in group membership and face memory; several other tasks were only associated with some measures of face recognition ability. These findings indicate that face perception deficits in DP may extend beyond identity perception. However, the associations between tasks may also reflect subtle aspects of task demands or stimuli.

Prosopagnosia is a condition characterised by severe difficulty identifying faces, in the absence of other major visual or cognitive deficits^1–3^. In some cases, prosopagnosia occurs following neurological illness or injury—this is referred to as acquired prosopagnosia (AP)^2^. In other cases, prosopagnosia occurs in the absence of any neurological damage. This form of the condition, known as developmental prosopagnosia (DP), affects roughly 2% of the adult population^4,5^ (although see^6^) and up to 4% of children^7^, and is thought to reflect a failure to develop the visuo-cognitive processes that underpin typical face recognition^3^.

While there is a host of research that has sought to examine which processes are impaired and intact in DP e.g.,^8–13^, there is still some disagreement as to whether DP should be considered a primarily perceptual deficit. In other words, it is unclear whether DP involves a difficulty constructing a typical percept of a face^11,14^, or whether some cases of DP have intact face perception, but instead reflect issues creating, storing, or accessing memories of faces^4,15^. Furthermore, if DP does reflect a perceptual deficit, there is substantial disagreement in the literature as to whether the deficit is specific to face identification, or whether it extends to other face perception tasks such as emotion recognition^16–21^, judgements of face age and gender^22–24^, and face detection^25^. In this study, we address these questions by examining whether different face perception tasks predict face identification abilities; specifically, whether identity-based tasks and non-identity based perceptual tasks predict how well individuals perform on face memory tasks, and whether they meet the diagnostic criteria for DP.

## Face identity perception in DP

The diagnostic protocols used in prosopagnosia research mean that, typically, individuals with DP show severe deficits on face memory tasks (e.g., famous face recognition tasks^15^; and/or the Cambridge Face Memory Task^26^). However, models of face recognition often distinguish between perceptual and mnemonic processes underpinning face recognition^27–29^, and impairments at either of these stages could result in difficulty with face memory tasks. Research showing variability in the presentation of DP supports the idea that face perception and face memory can be separably impaired: for example, Dalrymple et al.^30^ assessed 16 adult DPs on face memory and face identity perception, and found that only six showed significant impairments in face identity perception. Similarly, Bate et al.^31^ found face matching deficits (indicative of impaired face identity perception) in 15 out of 40 participants with DP, and Murray et al.^32^ found that almost half of their sample of individuals with DP (15/32) performed within the typical range on two forms of the Benton Face Recognition Test (the BFRTr^32^, and the BFRTc^33^), which tests face identity matching ability; this suggests that the remaining cases are likely to have difficulties with face memory as opposed to face identity perception. However, the conclusions in these papers were based on relatively conservative single case analyses or cut-off scores, which require relatively severe issues with face identity perception in order to reach significance.

Several recent studies have questioned the distinction between identity perception and mnemonic deficits in DP, finding evidence of relatively consistent, but subtle, perceptual deficits^8,11,14,34^. For example, Biotti et al.^14^ examined the performance of 72 participants with DP and 54 controls on the Cambridge Face Perception Test (CFPT^35^), a task requiring participants to sort morphed faces based on their resemblance to a (simultaneously presented) target face. Consistent with other work, only a small proportion of participants showed a severe impairment in the task (22/72 DPs scored more than 1.7 SD below the control mean). However, as a group, individuals with DP performed worse overall, and the authors noted that the pattern of performance was consistent with a shifted distribution for individuals with DP, rather than a small group of “apperceptive” cases lowering the mean for the DP group. Another recent study^8^ used performance on the CFPT to separate a group of 37 participants with DP into clusters. While the clusters differed in some measures of performance, it is notable that both clusters showed similarly poor performance in upright face identity perception tasks (the CFPT and a face matching task): that is, both clusters showed evidence of some perceptual deficit.

Further, there is evidence that some perceptual tasks (specifically, the CFPT and BFRTc) may predict the presence of DP relatively well. Mishra et al.^36^ examined the ability of these (and other) identity perceptual tests to predict DP diagnosis and performance on face memory tasks (the CFMT and a famous face identification task). Both the CFPT and BFRTc had relatively high sensitivity to DP (identifying true DP cases) and specificity (identifying true control cases), and in combination they significantly predicted the diagnosis of DP and scores on both face memory tasks. Notably, the other identity perception tasks that Mishra et al. examined were not significant predictors of DP diagnosis or of performance on face memory tests.

Mishra et al.’s findings lend some support to the idea that identity perception impairments are common in cases of DP, to the extent that face identity perception alone is a good predictor of the presence of DP. However, their findings also suggest that not all face perception tasks are sensitive to the deficits that may be present in DP. As such, it is important to examine which perceptual tests (if any) predict the presence of DP and/or performance on tests of face memory. On top of contributing to the debate around the pervasiveness of perceptual deficits in DP, this may have important implications for the development of diagnostic batteries, by broadening the range of tasks that are used to assess and diagnose face recognition deficits.

## Non-identity face perception in DP

Importantly, face perception encompasses more than simply identification. For example, most people can extract information about a person’s emotional expression, their age, and their gender from their face alone. Early models of face processing e.g.,^27,29^ proposed that some of these abilities were somewhat separable from identification. However, more recent studies have challenged the idea of a strict separation between identity and non-identity face processing in typical individuals (e.g., emotion and identity^37–39^), suggesting that the perceptual processes underpinning identity and non-identity processing are at least partially shared^40^. Consequently, if pervasive perceptual deficits are present in DP, it is possible that they extend to non-identity face perception tasks, as well as identity-based tasks.

Many studies have examined non-identity face perception in DP, with mixed findings. For example, Chatterjee and Nakayama^22^ examined age and gender perception in DP, using a sorting task designed to resemble the CFPT. A small subset of individuals with DP (5/19) showed a significant deficit in gender perception, there were no significant age perception deficits reported, and group-level comparisons did not reveal a difference between individuals with DP and the control group (see also^41,42^). However, in a recent study of gender perception, also using morphed images, Marsh et al.^24^ found poorer gender perception in DP across two different tasks (see also^19,23^). Similarly heterogeneous results appear in the emotion recognition literature: many studies report intact emotion recognition in DP eg.,^13^,^18^, while others report impairments in emotion recognition^17,19^. Biotti and Cook^17^ presented a group of individuals with DP with several challenging tasks of emotion recognition, and found significant deficits on the group level. More pertinently, though, performance on these emotion recognition tasks correlated with measures of face memory, suggesting that similar mechanisms could underpin deficits in emotion and identity in DP. Given the variability between studies (and, in some instances, within a single case^19^), it is possible that these discrepancies have arisen due to variation in the task demands and stimuli used in different studies.

To date, most studies have examined non-identity face perception in DP in a relatively domain-specific way (that is, examining different non-identity face perception tasks separately; e.g.,^17,24^), or in case study/case series designs (e.g.,^19,42^). Consequently, it is unclear whether some types of tasks are better predictors of face memory than others. This question is important given the limited number of existing tests that can be used in DP diagnosis: if the relationship between face memory and face perception impairments can be elucidated, face perception tasks may offer a novel range of screening tests that are rapid and simple to administer.

## Face detection in DP

Another important element of face perception is the ability to detect a face in the visual field. Face detection is often considered a separate stage in face processing, distinct from identification^43^. However, there is evidence that face detection relies on some similar perceptual cues as other face perception tasks: for example, face detection is associated with detection of specific facial features (specifically the eyes and mouth)^44^ and, like other face perception tasks, is negatively affected by inversion^25^. While this does not necessarily imply that face detection relies on the same perceptual mechanisms as other face perception tasks (i.e., the same holistic processing used for emotion and identity processing), if individuals with DP struggle to extract information about facial features and/or their configuration^8^, it is possible that these difficulties could also extend to face detection tasks. Indeed, some research has found that, on a group level, individuals with DP do show poorer face detection than controls^25^. However, other studies (often using more conservative criteria for detecting impairment) have observed typical face detection ability in cases of DP^19,42,45^.

## The current study

In this study, we were interested in whether DP is characterised by face perception deficits, and, if so, how broad those deficits are. To address this question, we examined whether various face perception tests could accurately predict a diagnosis of DP (indicating that impaired face perception is associated with the presence of DP in our sample), and if performance on those tests is associated with face memory more generally.

The first aim of this study was to replicate the findings presented by Mishra et al.^36^ in relation to face identity perception. Specifically, we used the same analytical techniques to examine how well two face identity perception tasks (in this case, the CFPT and a sequential matching task) predicted the presence of DP and performance on face memory tests. The second aim of this study was to extend these findings by examining whether other face perception tasks (those not involving identification) also predicted the presence of DP, as well as scores on individual face memory tasks.

Unlike previous work, our analyses incorporated tasks from multiple domains of face perception—face detection, emotion recognition, extraction of visually derived semantic codes (age and gender), and identification. As it is possible that some of the conflicting results found in the literature to date may be a result of idiosyncratic task demands or stimuli^17,22,36^, or relatively small samples^6^, we collected data for a sizable sample of individuals with DP, and included two tasks within each domain, each with different stimuli and/or task requirements. This allowed us to develop a broader understanding of face perception within DP.

## Methods

### Participants

The participants reported in this study are a subset of a wider group of individuals tested in our lab—the performance of these participants on a variety of tasks has also been reported in Bate, Bennetts, Tree, et al.^31^ and Bennetts et al.^8^. For ease of comparison across studies, the IDs for the DP participants reported in this paper and accompanying data are matched to the IDs used in Bennetts et al.^8^. The IDs for the DP participants whose data was also reported in Bate et al.,^31^ are also included in the accompanying datafile.

Thirty adults with DP (15 female; 15 male; age range = 18–73 years, M = 46.90, SD = 16.70) took part in this study. All participants were invited to our laboratory for an assessment, after contacting us via our website and reporting severe difficulties with face recognition. Subjective impairments were verified via a semi-structured cognitive interview, which examined difficulties with faces and other objects in daily life, the duration and severity of these difficulties, and any potential physiological cause of the difficulties (e.g., history of brain injury/illness, diagnosis of developmental disorders or severe visual impairment). Participants completed a battery of tests designed to assess their face processing, general visual processing, and general cognitive skills. The basic battery has been described in detail elsewhere^8,31^. However, in short, all participants with DP performed significantly (> 1.7SDs) below age-matched control cut-offs on the Cambridge Face Memory Test (CFMT^26^) (see below for details of age-matched control groups) and a famous faces test^15^. No participants reported a history of socio-emotional, psychiatric or neurological disorders, but five potential DP participants were excluded due to scoring above 32 on the Autism Quotient^46^. None of the participants with DP were excluded on the basis of their general cognitive performance: all participants had an estimated IQ > 70 based on the Wechsler Test of Adult Reading (WTAR^47^; and no participant over the age of 65 years scored less than 26 on the Mini Mental State Examination^48,49^. No DP participants showed pervasive difficulties with lower level vision or object categorisation, assessed via a Snellen letter chart (3 m), the Hamilton-Veale contrast sensitivity test), and five sub-tests of the Birmingham Object Recognition Battery (BORB^50^: Line Match, Size Match, Orientation Match, Position of the Gap Match, and Object Decision Test (hard version). A summary of each DP participants’ performance on the diagnostic battery is shown in Table 1.

**Table 1:** DP participants’ scores on screening measures, with DP and age-matched control group mean scores.

Participant ID	Age	Gender	CFMT (/72)	Famous face (% correct)	BORB: Length (/30)	BORB: Size (/30)	BORB: Orientation (/30)	BORB: Gap (/40)	BORB: Object Decision (/64)
DP01	18	M	42	61.67	28	27	30	45	55
DP02	19	F	39	56.25	24	25	25	33	55
DP04	21	M	33	57.58	26	27	20	31	49
DP05	23	M	41	75.00	28	24	25	35	56
DP06	24	M	40	65.00	24	27	25	36	56
DP08	26	F	40	47.37	27	28	26	37	58
DP09	29	F	33	38.89	27	28	25	37	56
DP11	33	M	30	48.33	28	29	29	30	53
DP12	34	M	36	51.67	25	23	27	32	51
DP13	37	F	33	49.12	26	28	25	37	58
DP14	37	M	32	50.00	29	27	26	31	60
DP18	46	F	35	18.75	27	29	28	38	58
DP21	49	M	39	73.68	24	25	23	33	52
DP22	50	F	29	46.67	26	26	25	24	52
DP23	52	M	33	31.03	28	29	28	37	59
DP24	52	F	37	61.02	26	26	25	37	55
DP25	53	F	33	48.33	25	24	26	40	56
DP28	54	M	35	51.79	28	27	27	39	57
DP30	56	F	41	32.69	28	24	26	36	57
DP31	56	F	41	41.38	28	28	26	38	56
DP32	58	F	28	44.07	27	28	25	29	57
DP33	59	M	29	23.64	26	29	26	37	53
DP35	62	M	37	50.00	24	26	25	32	46
DP36	63	F	37	66.04	27	27	25	36	49
DP40	65	M	39	71.67	29	25	28	36	55
DP41	66	F	38	56.00	23	26	27	35	53
DP44	67	F	38	34.62	30	25	27	39	59
DP45	68	M	41	49.15	28	26	29	38	61
DP46	73	F	39	35.56	28	24	22	29	49
DP50	57	M	38	76.67	27	28	23	36	56
DP group (N = 30)	46.90 (16.70)	15 F	36.20 (4.06)	50.46 (14.71)	26.70 (1.74)	26.50 (1.74)	25.80 (2.11)	35.10 (4.10)	54.90 (3.58)
Controls (younger) (N = 49)	32.41 (9.16)	24 F	57.49 (8.49)	92.30 (7.03)	26.57 (1.55)	27.88 (1.39)	26.08 (2.50)	36.92 (2.16)	55.82 (3.22)
Controls (older) (N = 26)	59.96 (6.81)	11 F	57.04 (8.87)	90.99 (8.36)	26.46 (1.94)	27.58 (1.77)	25.92 (2.77)	36.12 (2.39)	55.23 (3.87)

CFMT: Cambridge Face Memory Test^26^; BORB: Birmingham Object Recognition Battery^50^. IDs for DP participants match those used in Bennetts et al.^8^.

A total of 75 control participants (35 female, age range = 20–73 years, M = 41.96, SD = 15.63) also took part in this study. An additional 27 control participants took part in the study, but their data had to be excluded due to missing tasks (19 participants) or as outliers (> 3SD from age-matched control mean on any one task or condition; 8 participants). No individual reported everyday difficulties in face recognition, and all controls performed within the typical range (< 2 SDs from the age-matched mean) for the CFMT and famous faces task, based on age-matched norms from Bate et al.^15^ (three controls did not complete the famous faces task; however, all performed in the typical range for the CFMT and CFPT, and were therefore retained in the sample). The control group did not report any history of socio-emotional, psychiatric or neurological disorders. All controls scored within the typical range in the AQ, WTAR, and BORB (between 1 and 4 control participants did not complete these tasks; however, none reported any visual deficits or showed abnormal performance on any other measure, therefore all were retained in the sample).

Although we endeavoured to match the groups as closely as possible on demographic and screening variables, independent samples *t*-tests revealed that there were small but significant differences between the control and DP groups in the WTAR, BORB (size) and BORB (position of gap) tasks; WTAR: M_DP_ = 116.97, SD = 5.30; M_CONTROL_ = 112.81, SD = 8.02, *t*(103) = 2.61, *p* = 0.01; BORB; size: M_DP_ = 26.50, SD = 1.74; M_CONTROL_ = 27.77, SD = 1.53, *t*(103) = 3.71, *p* < 0.01; position of gap: M_DP_ = 35.10, SD = 4.11; M_CONTROL_ = 36.64, SD = 2.26, *t*(103) = 2.46, *p* = 0.02. No other comparisons were significant.

Controls were recruited from the departmental participant pool, and received a small financial payment in exchange for their time. For the purpose of calculating norms, and as previous work has suggested minimal decline in face recognition abilities prior to 50 years of age^4^, the control group was split into two age groups: 20–49 yrs (N = 49, 11 F, 15 M, M_age_ = 32.41, SD = 9.16) and 50–73 (N = 26, 24 F, 25 M, M_age_ = 59.96, SD = 6.81). The CFMT and famous faces scores for these age groups were used as the basis for calculating impairment for the DP participants.

Ethical approval for this experiment was granted by the departmental ethics committee at Bournemouth University, and all experiments followed relevant ethical guidelines and regulations. All participants provided informed consent according to the Declaration of Helsinki.

## Materials

Participants completed eight tasks assessing various aspects of face perception: identity perception; emotional expression recognition; non-identity semantic information (age and gender); and face detection.

### Face identity perception tasks

#### *Cambridge face perception test (CFPT)*^35^

The CFPT is a standardised test of face perception. In each trial, participants are shown one target face at the top of the screen, and six comparison faces at the bottom of the screen. Each of the comparison faces has been morphed to resemble the target face to a different degree. Participants are asked to sort the comparison faces based on their similarity to the target face. Each trial lasts up to 60 s.

The CFPT contains eight upright and eight inverted trials, presented in a fixed semi-random order). Only scores for the upright trials were used in the current analyses. Performance is scored by summing deviations from the correct order (e.g., if a face is three spaces from its correct location, it would add three to the deviation score), so that a higher score equates to worse performance. To aid in the analysis, we converted participants’ raw scores into percentage correct using the formula [100 x (1-(deviation score/maximum score))] as per Rezlescu et al.^51^.

#### *Face matching*^31,52^

This task uses a sequential matching procedure, where participants are asked to indicate if two faces (presented one after the other) show the same identity or two different identities. In each trial, participants view a single, frontal image of a face for 250 ms; followed by a 1000 ms ISI, then a second, slightly angled (around 30 degrees) image of a face (presented until a response was recorded). Participants respond via keypress (assignment of response keys was counterbalanced between participants). Participants are instructed to respond as quickly and as accurately as possible.

The task includes 64 upright and 64 inverted trials (16 same identity pairs; 16 different identity pairs, each presented twice). Participants complete the same matching task with three different object categories (faces, hands, and houses), with the presentation of object categories blocked and randomised between participants. Within each block, the order of trials is randomised. Each block is preceded by six practice trials (containing different stimuli to the main experiment).

For the current analysis, only upright face trials were analysed. Accuracy was calculated separately for same-identity (hits) and different-identity (correct rejections) trials in each condition, and combined into a bias-free measure of sensitivity, *d’*^53^ and bias (c). Reaction times were also collected, but they were not analysed as part of the current study.

### Emotional expression recognition

#### *Ekman 60 faces*^54^

In this task participants are asked to categorise pictures of actors as one of six basic emotions: anger, happiness, sadness, fear, surprise, or disgust. The stimuli consist of pictures of ten actors (four male and six female), which are displayed on screen for five seconds each. Participants indicate their response by a mouse click on the relevant button onscreen. There is no time limit for responses. There are 60 trials in total in the task.

#### *Reading the mind in the eyes test (RMITE)*^55^

In the RMITE test, participants are presented with greyscale images of the eye region of actors (males and females), accompanied by a list of four mental states (the mental state terms vary from trial to trial). Participants are asked to select the term which best describes what the person onscreen is thinking or feeling. Images are presented on screen for an unlimited amount of time, and responses are indicated by a key press. Although the RMITE task was originally devised as a theory of mind task, more recent work has regarded it as a test of subtle emotion recognition^56^. Unlike the Ekman 60 faces task, which only includes six basic emotions, the RMITE task includes more complex emotions and mental states (e.g., hostile, irritated, pensive, flirtatious). There are 36 trials in the task, preceded by one practice trial.

### Visually derived semantic codes

#### *The philadelphia face perception battery (PFPB)*^57^

Two subtests from the PFPB were used to assess age and gender perception. In the 75 trials of the age subtest of the PFPB, participants are presented with two faces onscreen simultaneously, and asked to select which one appears older by clicking on the corresponding image. In the gender subtest, participants are presented with 75 faces, one at a time, and asked to indicate whether the person is male or female by clicking on the matching button onscreen. In both subtests, the faces are frontal, colour images of computer-generated Caucasian faces, cropped to remove the hair (although the ears and jawline remain visible). During test development, trials were selected to be of moderate difficulty (minimum 80% cross-participant agreement on the correct response), and in the final version, trials are presented in ascending order of difficulty^57^.

### Face detection

#### Scrambled scenes

**Figure 1:**
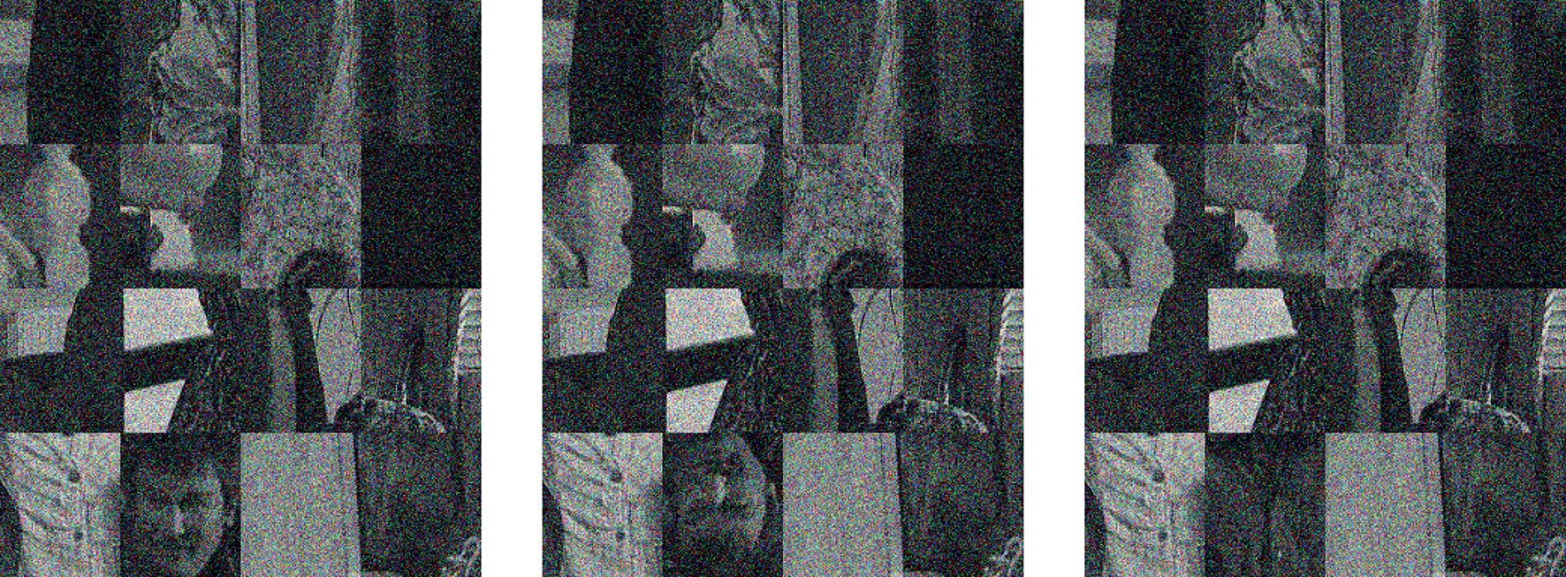
Examples of the scrambled scenes stimuli. From left to right: target present (upright face); target present (inverted face); target absent. The face image in the first two examples is present in the bottom row, second tile from the left.

This is a new task, created in our lab – the design is based broadly on the Face versus Non-Face task presented in Garrido et al.^25^, but new stimuli were used in this study. In this task, participants are asked to indicate the presence or absence of faces in images extracted from scenes. In each trial, 16 tiles are presented in a 4 × 4 configuration onscreen (see Fig. 1). Each tile contains a section of scene depicting indoor and outdoor environments, objects, and, in some cases, people. The images were taken as screenshots from a Welsh television show, which was intended to be unfamiliar with participants. To create each trial, images were converted to greyscale and overlaid with visual noise (a Gaussian blur of 15%), then split into rectangular tiles, the order of the which was scrambled. In half of the trials, one tile depicted an image of a face (presented wholly within one tile and filling the majority of the tile). Faces in this task are naturalistic—there is some variation in viewpoint (from frontal to roughly a 45-degree angle) and expression, and the images included hair. The images are presented once with the face upright and once with the face inverted. Each condition (upright and inverted) includes 15 face-present trials and 15 face-absent trials (as only the face was changed in inverted trials, the target-absent trials are identical for upright and inverted scenes). Upright and inverted trials are blocked, with upright trials presented first. Trial order is randomised within each block.

#### *Two-tone face detection*^25^

The second face detection test required participants to indicate whether a “two-tone” face—that is, a face that has been filtered and thresholded to create a line drawing—is present or absent in a display of scrambled two-tone face parts. As in the scrambled scenes task, each display in the task is presented once upright and once inverted. Each condition (upright and inverted) contains 36 face-present trials and 12 face-absent trials, and the conditions were blocked, with upright trials presented first.

In both tasks, participants have a maximum of 8 s to indicate whether the image contains a face; images remain on-screen until participants make a response or the trial times out.

Results for the upright condition only were included in the current analysis. For both detection tasks, accuracy was calculated separately for face-present (hits) and face-absent (correct rejections) trials, and combined into a bias-free measure of sensitivity, *d’* and bias (c) (Macmillan & Creelman, 2005). Mean reaction times for correct trials (excluding any RTs below 150 ms or above 4000 ms) were also calculated for each condition in each task.

## Results

Descriptive statistics and reliability for each group of participants in each task are presented in the Supplementary materials (Tables S1 and S2).

### Analytical approach

Similar to Mishra et al.^36^, we examined the predictive power of different perceptual tests in several ways: first, we examined whether the control and DP groups showed significant differences in performance in each task using one-way ANCOVAs, with group as a between-subjects factor and age as a covariate. Next, we used logistic regressions to analyse how effectively different perceptual tests or measures predicted DP or control group membership, alone or in combination with other tests. Finally, we used linear regressions to examine how the same measures of face perception predicted objective measures of face recognition—specifically, scores on the CFMT and famous faces tasks. Including both logistic and linear regressions in the analyses allowed us to examine both the utility of different face perception measures for identifying severe face recognition impairments (logistic regressions), and also to determine whether those same tasks predict performance across the full spectrum of face recognition abilities (linear regressions).

In the initial analyses, we entered the identity perception tests (CFPT and matching test) alone; subsequently, we included the remaining (non-identity) face processing tasks from the battery. Non-identity tasks were analysed both with and without the identity-based face perception tasks included in the model, to examine (a) whether they are able to predict DP on their own, and (b) if they account for unique variance above and beyond the identity perception tasks. Age was included in all regressions as part of the null model. All reported beta values are unstandardised betas. Full coefficient tables for all analyses are presented in the Supplementary materials (Tables S3-S5).

All analyses were carried out in JASP (v 0.16.), and the data associated with the study can be accessed at https://osf.io/va4jh/.

### Predicting face recognition ability from face identity perception tests

**Figure 2:**
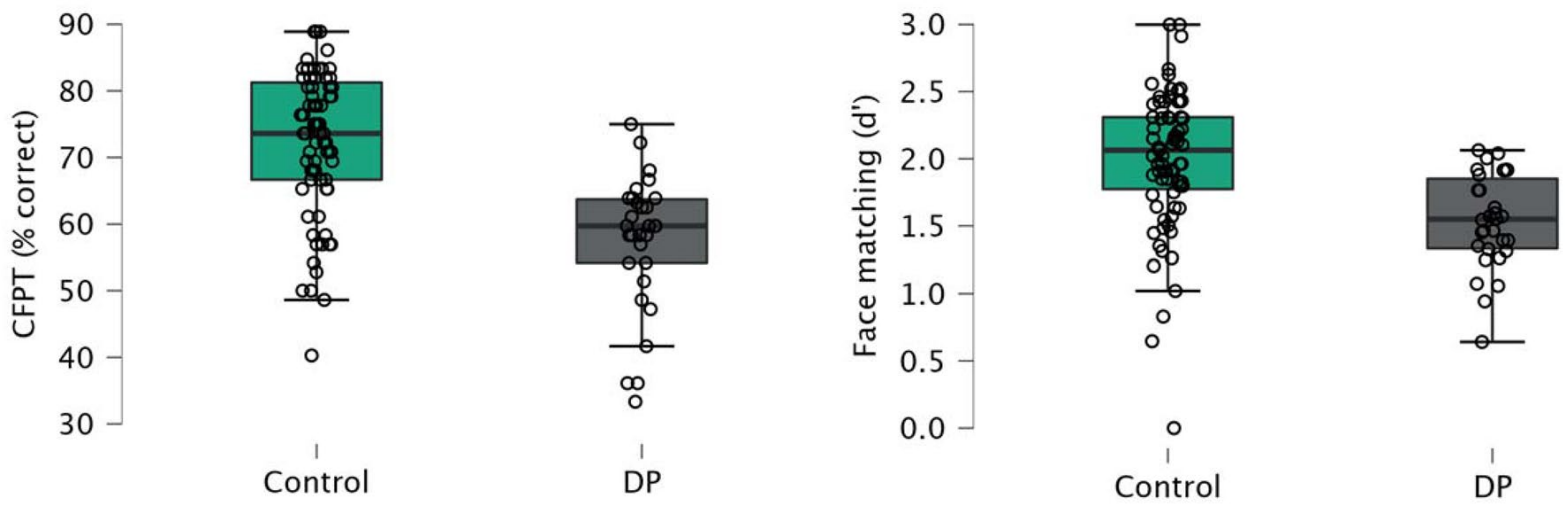
DP and control participant performance on face identity perception tasks.

Performance of the DP and control groups on the face identity perception tasks (the CFPT and the matching task) are shown in Fig. 2. ANCOVAs on each task confirmed that the DP group scored significantly lower than the control group on average in both tasks; CFPT: *F*(1,102) = 40.00, *p* < 0.001, η^2^_p_ = 0.28; matching: *F*(1,102) = 19.12, *p* < 0.001, η^2^_p_ = 0.16.

A logistic regression with the CFPT and matching tasks both entered as predictors, age included in the null model, and DP or control group membership as the outcome variable showed a significant model fit, *χ*^2^ (101) = 37.30, *p* < 0.001, AIC = 94.28, Nagelkerke *R*^*2*^ = 0.43. Classification accuracy was high (77.1%; compared to 71.4% for the null model) and ROC analyses revealed that the model showed excellent specificity (0.88) but poor sensitivity (0.50) to DP. Both the CFPT and the matching task predicted unique variance in DP/control group categorisation: CFPT: *b* = 1.33, Odds Ratio = 3.77 [95% CI 1.91, 7.46], *p* < 0.001; matching task: *b* = 0.69, Odds Ratio = 2.00 [95% CI 1.06, 3.77], *p* = 0.033.

A linear regression with both the CFPT and matching tasks as predictors, age included in the null model, and the CFMT as an outcome variable was significant, *F*(2,101) = 42.45, *p* < 0.001, *R*^*2*^ = 0.45. Both the CFPT and the matching task predicted unique variance in CFMT scores, CFPT: *b* = 5.51 [95% CI 3.51, 7.50], *t*(101) = 5.48, *p* < 0.001; matching task: *b* = 4.47 [95% CI 2.41, 6.53], *t*(101) = 4.30, *p* < 0.001. An identical regression with the famous faces task as the outcome variable was also significant overall, *F*(2,100) = 17.34, *p* < 0.001, *R*^*2*^ = 0.25; and again, both the CFPT and the matching task predicted unique variance in famous faces scores, CFPT: *b* = 8.04 [95% CI 3.98, 12.11], *t*(100) = 3.93, *p* < 0.001; matching task: *b* = 4.68 [95% CI 0.48, 8.89], *t*(100) = 2.21, *p* = 0.030.

### Predicting face recognition ability from non-identity face perception tasks

**Figure 3:**
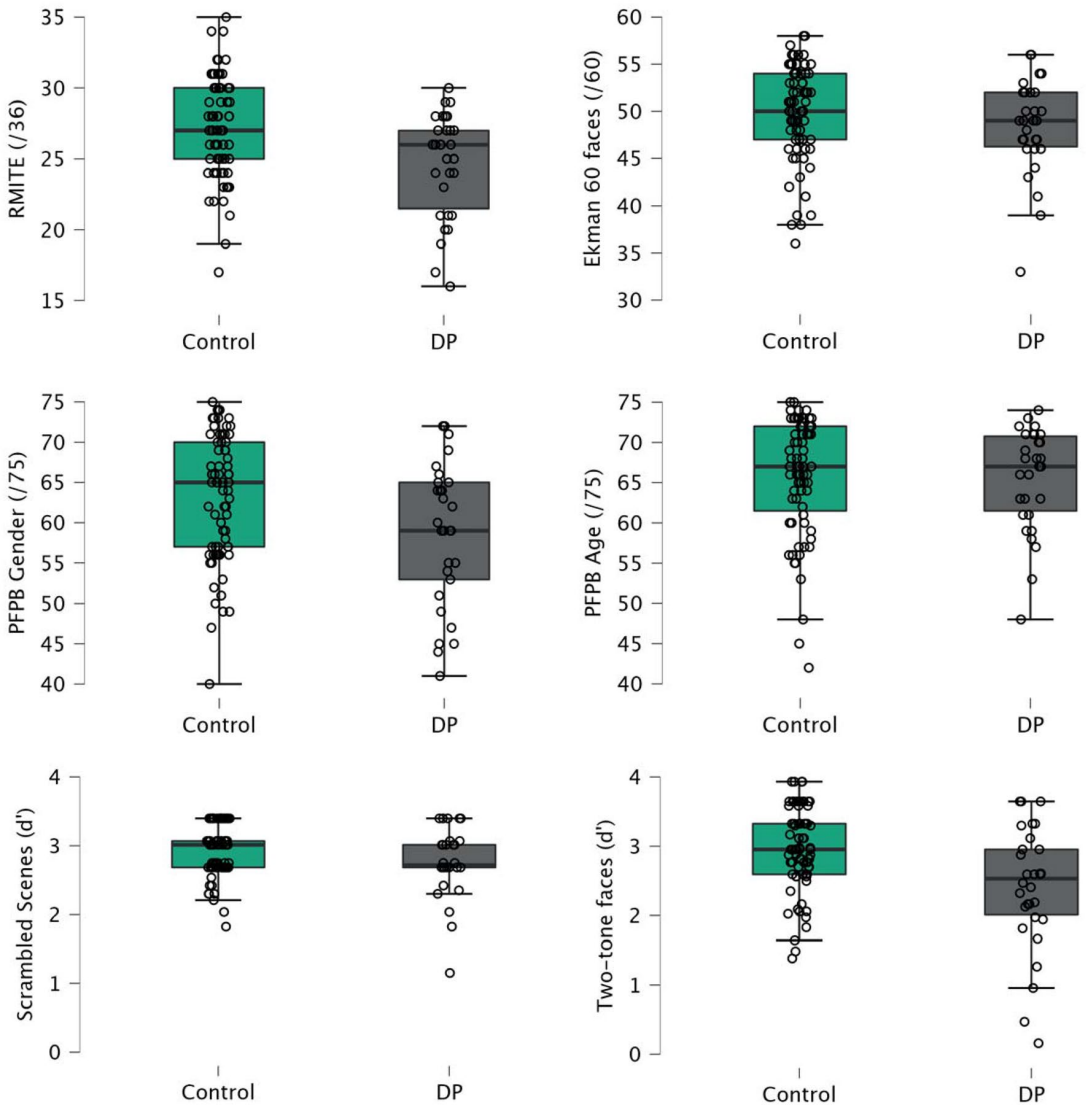
DP and control participant performance on non-identity face perception tasks: emotion recognition (top row); visual semantic information (middle row); face detection (bottom row).

Performance of the DP and control groups on the non-identity face perception tasks (emotion, visual semantic information, and face detection tasks) is shown in Fig. 3. ANCOVAs on each task showed a significant difference between control and DP groups for the RMITE (*F*(1,102) = 10.75, *p* = 0.001, η^2^_p_ = 0.10), PFPB gender (*F*(1,102) = 6.90, *p* = 0.010, η^2^_p_ = 0.06), and two-tone face detection (*d’*) (*F*(1,102) = 14.00, *p* < 0.001, η^2^_p_ = 0.12) tasks. The Ekman 60 faces, PFPB age, and scrambled scenes (*d’*) tasks did not show significant differences between groups, all *p*’s > 0.08.

To examine whether non-identity based face perception tasks were able to predict DP or control group classification, we carried out a logistic regression with all six non-identity face tasks as predictors, age included in the null model, and DP or control group membership as the outcome variable. The results showed a significant model fit, χ^2^ (97) = 24.89, *p* < 0.001, AIC = 114.70, Nagelkerke *R*^*2*^ = 0.30. Classification accuracy was identical to the model with the identity-based tasks (77.14%), and, similarly to that model, ROC analyses indicated excellent specificity (0.92) but poor sensitivity (0.40) to DP. Performance on the PFPB gender task and the two-tone face detection task predicted unique variance in DP/control group categorisation: PFPB gender: *b* = 0.64, Odds Ratio = 1.90 [95% CI 1.11, 3.27], *p* = 0.019; two-tone face detection: *b* = 0.80, Odds Ratio = 2.23 [95% CI 1.24, 4.02], *p* = 0.008. None of the other tasks accounted for significant unique variance, all *p*’s > 0.075.

A linear regression with all six non-identity based face perception tasks as predictors, age included in the null model, and the CFMT as the outcome variable, was significant, *F*(6,97) = 5.16, *p* < 0.001, *R*^*2*^ = 0.24. Only the PFPB gender task and the two-tone face detection task predicted unique variance in CFMT scores, PFPB gender: *b* = 2.52 [95% CI 0.20, 4.84], *t*(97) = 2.16, *p* = 0.033; two-tone face detection: *b* = 2.88 [95% CI 0.45, 5.32], *t*(97) = 2.35, *p* = 0.021. An identical regression with the famous faces task as the outcome variable was also significant overall, *F*(6,96) = 4.52, *p* < 0.001, *R*^*2*^ = 0.21. Once again, the PFPB gender task predicted unique variance, along with the RMITE task, PFPB gender: *b* = 5.20 [95% CI 1.06, 9.34], *t*(96) = 2.49, *p* = 0.014; RMITE: *b* = 5.90 [95% CI 1.54, 10.27], *t*(96) = 2.68, *p* = 0.009.

Next, we analysed whether any of the non-identity based face perception tasks accounted for unique variance in DP or control group classification, after accounting for the identity-based perception tests. First, we conducted a two-block analysis designed to examine whether non-identity tasks significantly predicted face recognition ability once the variability associated with identity perception tasks had already been accounted for—in other words, are non-identity tasks providing predictive power above and beyond what is already present in identity tasks. A logistic regression with all six non-identity face tasks as predictors, DP or control group membership as the outcome variable, and the CFPT, matching tasks, and age included as part of the null model showed a significant model fit, χ^2^ (91) = 22.19, *p* = 0.001, AIC = 81.80, Nagelkerke *R*^*2*^ = 0.35. Classification accuracy was very high (86.1%), and ROC analyses revealed that the model showed excellent specificity (0.90) and very good sensitivity (0.77) to DP. Performance on the PFPB age task, the PFPB gender task, and the two-tone face detection task predicted unique variance in DP/control group categorisation: PFPB age: *b* = −1.52, Odds Ratio = 0.22 [95% CI: 0.07, 0.64], *p* = 0.006; PFPB gender: *b* = 1.02, Odds Ratio = 2.78 [95% CI: 1.24, 6.27], *p* = 0.014, two-tone face detection: *b* = 0.97, Odds Ratio = 2.64 [95% CI: 1.24, 5.65], *p* = 0.012. None of the other tasks accounted for significant unique variance, all *p*’s > 0.09.

A linear regression with the identity perception tasks (and age) entered as the first block and the non-identity tasks entered as the second block showed that the non-identity tasks did not predict a significant amount of variance in CFMT scores once the identity tasks were accounted for, *F*(6,95) = 2.17, *p* = 0.052, *R*^*2*^ change = 0.06. However, if all variables (including identity tasks) were entered as a single block, the PFPB gender task still predicted unique variance in CFMT scores, *b* = 1.94 [95% CI: 0.07, 3.81], *t*(95) = 2.06, *p* = 0.042. The same linear regression with famous face recognition scores as an outcome was significant, *F*(6,94) = 3.77, *p* = 0.002, *R*^*2*^ change = 0.14. In this analysis, scores on the RMITE, PFPB gender, and PFPB age tasks all predicted unique variance in famous face recognition; RMITE: *b* = 5.01 [95% CI: 1.13, 8.89], *t*(94) = 2.56, *p* = 0.012; PFPB gender: *b* = 4.45 [95% CI 0.77, 8.13], *t*(94) = 2.40, *p* = 0.018; PFPB age: *b* = −5.75 [95% CI −10.22, −1.28], *t*(94) = —-2.55, *p* = 0.012.

### Additional analyses

Follow-up analyses were carried out to compare the relationship between face perception tasks and face memory tasks across the DP and control groups (see Supplementary materials, Tables S6 and S7), for all the linear regressions reported above. The linear regressions were conducted separately for each participant group, and the coefficients that were significant in the main analysis were then compared for the DP and control groups, to assess whether the relationship between tasks was different for individuals with and without face recognition difficulties. Across all the predictors that were significant in the main analyses, only one varied significantly between the DP and control groups. In the analysis which only included identity perception tests, when predicting CFMT scores, the face matching regression coefficients for the control group were significant, whereas those for the DP group were not; the difference between the regression coefficients across groups was also significant. In sum, face matching explains more unique variance in CFMT performance in participants without face recognition impairments, compared to those with DP.

Some previous work^25^ has indicated that reaction time measures are more sensitive to face recognition deficits than sensitivity measures; as such, the ANCOVAs and regression analyses which included the two-tone face detection and scrambled scenes tasks were rerun with RT instead of *d’*. The ANCOVAs indicated that control participants were significantly faster than DPs in the two-tone face detection task, M_DP_ = 2196.50, M_control_ = 1706.78, *F*(1,102) = 16.85, *p* < 0.001, η^2^_p_ = 0.14. There was no difference in RT between the control and DP groups, M_DP_ = 1738.53, M_control_ = 1655.79, *F*(1,102) = 0.014, *p* = 0.71, η^2^_p_ = 0.00.

All regression models including face detection RTs were significant (see Tables 2, 3 and 4). The results of the logistic regressions did not change substantially, except that the PFPB age task no longer predicted DP or control group membership. For the linear regressions, RT for two-tone face detection tasks was a significant predictor in all analyses, and performance on the Ekman 60 faces task was a significant predictor of CFMT scores when identity tasks were not included in the models. Conversely, performance in the PFPB gender and age tasks did not predict performance on either the CFMT or famous faces when RT measures of face detection were included in the analyses. The RMITE task remained significant in the models predicting famous face recognition performance. See Tables 2, 3 and 4 for regression model summaries, and Supplementary materials (Tables S8-S10) for full coefficient tables.

**Table 2:** Summary of logistic regression models predicting DP/control group membership.

Model	AIC	df	Χ^2^	p	Nagelkerke R^2^	Accuracy/ Sensitivity/ Specificity	Significant predictors (*b*)
1. Identity perception tasks	94.28	101	37.30	** < .001**	0.43	77.1%/ 0.50/ 0.88	CFPT (1.33) Face matching (0.69)
2. Non-identity face perception tasks	114.70	97	24.87	** < .001**	0.30	77.1%/ 0.40/ 0.92	PFPB gender (0.64) Two-tone faces (0.80)
3. Non-identity face perception tasks, controlling for identity tasks	81.80	91	22.19	**.001**	0.35	86.1%/ 0.77/ 0.91	PFPB age (-1.52) PFPB gender (1.02) Two-tone faces (1.82)
2a. Non-identity face perception tasks (RT)	106.30	97	33.27	** < .001**	0.39	80.95%/ 0.53/ 0.92	PFPB gender (0.63) Two-tone faces (-1.36)
3a. Non-identity face perception tasks, controlling for identity tasks (RT)	76.68	91	27.32	** < .001**	0.42	86.14%/ 0.73/ 0.92	PFPB gender (1.10) Two-tone faces (-1.65)

Participant age was included in the null model for all analyses. For models 3 and 3a: participant age, CFPT score, and face matching performance were included in the null model. Models 2a and 3a included RT instead of *d’* for the two face detection tasks; all other measures remained the same.

**Table 3:** Summary of linear regression models predicting CFMT performance.

Model	*R*^*2*^	RMSE	*R*^*2*^ Change	*F* Change	df1	df2	*p*	Significant predictors (*b*)
1. Identity perception tasks	0.468	9.033	0.447	42.447	2	101	**< .001**	CFPT (5.51) Face matching (4.47)
2. Non-identity face perception tasks	0.257	10.886	0.237	5.165	6	97	**< .001**	PFPB gender (2.52) Two-tone faces (2.88)
3. Non-identity face perception tasks, controlling for identity tasks	0.532	8.734	0.064	2.172	6	95	0.052	PFPB gender (1.94)
2a. Non-identity face perception tasks (RT)	0.31	10.46	0.29	6.94	6	97	**< .001**	Ekman 60 faces (2.83) Two-tone faces RT (-4.95)
3a. Non-identity face perception tasks, controlling for identity tasks (RT)	0.57	8.39	0.10	3.66	6	95	**0.003**	Two-tone faces RT (-3.59)

Participant age was included in the null model for all analyses. For models 3 and 3a: participant age, CFPT score, and face matching performance were included in the null model. Models 2a and 3a included RT instead of *d’* for the two face detection tasks; all other measures remained the same.

**Table 4:** Summary of linear regression models predicting famous face recognition performance.

Model	*R*^*2*^	RMSE	*R*^*2*^ Change	*F* Change	df1	df2	*p*	Significant predictors (*b*)
1. Identity perception tasks	0.286	18.30	0.25	17.34	2	100	**< .001**	CFPT (8.04) Face matching (4.68)
2. Non-identity face perception tasks	0.251	19.14	0.21	4.52	6	96	**< .001**	RMITE (5.90) PFPB gender (5.20)
3. Non-identity face perception tasks, controlling for identity tasks	0.425	16.95	0.14	3.77	6	94	**.002**	RMITE (5.01) PFPB age (-5.75) PFPB gender (4.45)
2a. Non-identity face perception tasks	0.35	17.77	0.32	7.80	6	96	** < .001**	RMITE (5.52) Two-tone faces RT (-9.79)
3a. Non-identity face perception tasks, controlling for identity tasks	0.50	15.80	0.21	6.69	6	94	**< .001**	RMITE (4.57) Two-tone faces RT (-7.98)

Participant age was included in the null model for all analyses. For model 3: participant age, CFPT score, and face matching performance were included in the null model. Models 2a and 3a included RT instead of *d’* for the two face detection tasks; all other measures remained the same.

Lastly, bias (c) was also subjected to the same one-way ANCOVAs as *d’* and RT for the scrambled scenes and two-tone face detection tasks. Neither analysis was significant: Scrambled scenes: M_DP_ = 0.09, M_control_ = −0.02, *F*(1,95) = 3.78, *p* = 0.055, η^2^_p_ = 0.04; two-tone face detection: M_DP_ = 0.13, M_control_ = 0.02, *F*(1,93) = 1.44, *p* = 0.232, η^2^_p_ = 0.02. As bias was not directly related to the hypotheses for this paper, no further analyses were carried out.

## Results summary

The models tested in each section of the results are summarised in Tables 2, 3 and 4. Our results indicated that both the CFPT and the facial identity matching task predicted significant unique variance in DP group membership (Table 2), CFMT scores (Table 3), and famous face recognition (Table 4). Follow-up analyses suggested that face identity matching may be a better predictor of CFMT scores for individuals with typical face recognition abilities compared to the DP group.

The pattern of results for non-identity face perception tasks was more complex, and varied somewhat depending on whether *d’* or RT measures of performance were included for the face detection tasks. In *d’* based analyses, scores on the PFPB gender task predicted unique variance in DP group classification, CFMT scores, and famous face recognition, even when identity perception tasks were accounted for. In addition, the two-tone face detection task predicted unique variance in DP group classification and CFMT scores. However, when identity perception tasks were added into the *d’* based models, the two-tone face detection effect only remained significant for group classification and not CFMT scores. The RMITE task was able to predict performance on the famous faces task, even after identity perception tasks were included in the model. Unlike the other tasks in the battery, the PFPB age task negatively predicted DP group classification and famous face recognition only once identity perception tasks were accounted for. Finally, the Ekman 60 faces task and the scrambled social scenes task did not contribute unique variance to any of the *d’* based models, nor were there significant differences in performance between the DP and control groups when the tasks were analysed separately. In contrast to this, models which included RT for the face detection tasks found that RT in the two-tone face detection task consistently predicted performance in the face recognition tasks, and the Ekman 60 faces task predicted CFMT performance when identity-based tasks were not included in the model. The RMITE task remained a significant predictor of famous face recognition performance.

While it is unsurprising that facial identity perception tasks were the strongest predictors of face memory performance (and hence DP membership given these tasks are core diagnostic indicators), it is pertinent that at least some measures of non-identity perception also contributed to the models. These effects were inconsistent across tasks, likely due to differences in task design and reliability (the non-identity tasks are generally less sophisticated than their identity counterparts), but we nevertheless can speculate that more generalised differences in face perception ability may underpin deficits in face recognition/memory. This would coincide with performance at the structural encoding phase of classical face recognition models (e.g.,^27^), and is also compatible with more recent neurological models that predict a common, core stage of early face perception (e.g.,^28,29^). While these models predict healthy and lesioned face recognition performance, the findings reported here suggest they are also compatible with developmental deficits in face recognition performance, such as DP.

## Discussion

This study examined whether a variety of face perception tests predicted face recognition performance—either categorically (distinguishing between participants with and without a diagnosis of DP) or continuously (predicting scores on face recognition tasks).

Both of the face identity perception tests included in the study (the CFPT and a sequential face matching task) predicted unique variance in DP group membership and continuous measures of face memory performance (CFMT scores and famous face recognition). This is broadly in line with the findings of Mishra et al.^36^, in that face perception tasks have some predictive power for categorising DP and face recognition performance more broadly. Unlike that study, though, our results indicate that both tasks may be sensitive to DP diagnosis and broader differences in face perception ability, as both account for unique variance in predicting these factors. Notably, unlike Mishra et al.^36^ we did not include the Benton Face Recognition Test in our battery, so we cannot conclude whether our results reflect differences between samples or differences in the tests themselves. We also note that the matching task analysed in our study was a better predictor of continuous measures of face memory (specifically CFMT scores) for individuals with typical face recognition ability than for individuals with DP. This may be a reflection of the smaller sample size in our DP group than the controls, or it may indicate that the face matching test is simply more sensitive to variation in the typical population. Nonetheless, the face matching task was a significant predictor when categorising DP and control participants; as such, our findings offer converging evidence that face perception tasks may have significant diagnostic utility when identifying cases of DP. This may be particularly useful when considering borderline cases, where face memory scores fall around diagnostic cut-offs. Currently, many laboratories exclude face perception tasks from their core diagnostic batteries, and the findings here suggest they may offer valuable supplementary information.

These findings lend some support to the idea that more generalised face perception deficits may be present in the majority of cases of DP (e.g.,^8,14^), although it is important to note that many of the participants with DP performed within the “typical” range on both face identity perception tests (i.e., < 1.7 SDs below the control mean). Based on our current findings, it is not possible to determine the nature of these perceptual deficits—for example, whether the deficits reflect issues with holistic or featural processing^8^; whether they are specific to faces or reflect more domain-general processes^31^; or whether these factors vary between individuals. Given previous work indicating substantial heterogeneity in the perceptual underpinnings of DP (e.g.,^8,31,42,45,58^), along with substantial overlap in the perceptual processes underpinning identity and non-identity face processing tasks^13,17,23,24^, we suggest that the current findings may represent a range of perceptual deficits (i.e., different “subtypes” of DP), as opposed to a single impaired mechanism affecting all participants. However, as the number of participants with DP is already modest for logistic regression analyses, we were unable to examine whether the perceptual tasks in our battery were equally predictive of different DP subtypes that have been identified in previous work (e.g.,^8,31^). Future work with larger samples may be able to address these questions, and identify whether certain identity and non-identity tasks are particularly effective at identifying specific types of perceptual deficits in DP.

Although the overall findings from the non-identity battery are mixed, the gender subtest from the PFPB predicted face recognition performance across all categorical and several continuous analyses (with the exception of models incorporating RT). On the one hand, this is unsurprising, as several studies have suggested that gender and identity perception rely on similar perceptual mechanisms (i.e., holistic and configural perception; see^41^, c. f.^59^), and that gender perception may be impaired in DP^23,24,60^ (although see^22,41^). However, if both tasks relied on identical perceptual and cognitive mechanisms, we would not expect gender perception to predict face recognition ability once identity perception was accounted for. Therefore, our findings suggest that there may be some mechanisms or cues that are used during gender perception tasks and face memory tasks, that are less relevant in tasks with a minimal memory component (such as the CFPT and matching tasks)—for example, matching a stimulus to a stored internal “template” or “norm”^61,62^. Further, our findings suggest that these mechanisms may also be impaired in DP (see also^63^). Notably, this raises the question of whether a gender matching or same/different task (which may rely on perceptual mechanisms more analogous to the unfamiliar face matching tasks used in this study) would also show strong associations with face memory, independent of face perception.

Reaction time in the two-tone face detection task also consistently predicted face recognition across both categorical and continuous analyses, indicating that there is a link between the detecting faces in scenes and identifying them. When combined with previous work showing that performance on both tasks is impaired by inverting the stimuli, these findings suggest that it is possible that both tasks rely on overlapping perceptual processes^25^. Nonetheless, the current findings are somewhat surprising as previous findings relating to DP and face detection have been mixed (e.g., see^19,25,45^). It is possible that the specific format of this task—namely, presenting a face amongst many distractor face parts—might explain the current findings. There is some evidence that a substantial proportion of individuals with DP experience difficulty processing face parts^8^, which, if it extends to detection tasks, could lead to slower performance in tasks requiring processing of multiple face parts. Further work developing non-identity face processing tasks which are sensitive to individual differences in different underpinning processes could help to clarify when and why certain tasks from different domains correlate, while others do not.

Several other measures and tasks (notably sensitivity in the two-tone face detection task and the RMITE) also contributed unique variance in some analyses, but their effects were inconsistent—for example, the RMITE task only explained unique variance in famous face recognition, performance on the two-tone face detection task (*d’*) only explained variance in DP classification and CFMT scores. The finding that different tasks predicted different measures of face memory supports the idea that there may be substantial differences between the face memory tasks commonly used to assess face recognition ability^15^, and research into DP may need to explicitly discriminate between factors, mechanisms, and tasks which predict one’s ability to learn a new face and recall it after a short delay (the CFMT), and the ability to recognise and link semantic information to already-familiar faces (famous face recognition tasks).

Several tasks (notably the Ekman 60 faces task and the scrambled scenes face detection task) did not discriminate between DP and control participants; this is in line with Palermo et al.^13^ and accuracy measures from Garrido et al.^25^. Further, detection of faces in scrambled scenes did not predict face performance in any face recognition task included in our analyses, and the Ekman 60 faces task was only predictive of CFMT scores in one analysis (when RTs for face detection tasks were incorporated into analyses). The finding that only one task in each of the non-identity based domains had a consistent relationship with face recognition ability is unlikely to reflect general statistical limitations: no tasks in the battery showed ceiling or floor effects, and reliability for most of these tasks was in the high-excellent range, with the exception of the scrambled scenes face detection task, which showed poor reliability. However, the reliability reported for this task is not out of line with the findings from other studies that have examined reliability in face processing within DP (e.g., Esins et al.^23^ reported poor reliability in several of the tasks in their battery), and may reflect the relatively small sample size for reliability calculations.

Rather than statistical limitations, the lack of consistency within domains suggests that some of the tasks may lack sensitivity to detect subtle deficits. This is in line with findings reported by Duchaine et al.^19^: the DP participant Edward showed relatively typical performance on the Emotion Hexagon task (which uses similar stimuli and response categories to the Ekman 60 emotions task), but extremely poor performance on the RMITE task. The results of the RT-based analyses—specifically the finding that RT in the two-tone face detection task explained unique variance for all the outcome measures—suggests that, in some cases, using alternate measures of performance might increase our ability to detect subtle deficits and relationships between tasks. However, this is not the case with all tasks—neither RT or *d’* for the scrambled scenes task explained any unique variance in any of the models.

Consequently. it is also possible that some of the effects may be highly task- or stimulus-dependent, as opposed to reflecting performance in the domain more generally. For example, several of the tasks which showed significant relationships with the CFMT—specifically the CFPT, the face matching task, and the PFPB tasks—present faces in a similar format (i.e., heavily cropped to remove external features, and with limited variation in colouring), and these relationships might partially reflect proficiency with processing these somewhat artificially edited images (although it is important to note several of these tasks also predict performance in the more naturalistic famous faces task). Likewise, the relationship between the RMITE task and famous face recognition may reflect the fact that both tasks use fairly naturalistic stimuli, rather than a relationship between emotion recognition and familiar face recognition. Alternatively, the RMITE-famous faces relationship might reflect the fact that both tasks involve processing relatively complex semantic information: identifying a familiar/famous face requires the retrieval names or other unique semantic information, and success on the RMITE requires semantic processing of complex emotion words. These subtle differences between stimuli and task demands may explain some of the inconsistent results within the DP literature in relation to emotion processing, gender classification, and face detection^17,18,22,24,25,42^.

Supporting the point that some of the relationships between DP and face recognition that exist in the literature are likely task-dependent, the PFPB age subtest showed an unusual pattern of results in this study, only explaining significant variance once identity perception was already accounted for. Notably, the direction of effect for the age subtest was opposite to all other tasks included in the models, indicating that these effects may not reflect the same underpinning mechanisms as those related to other tasks. It is likely these findings reflect some idiosyncratic elements of the task or stimuli. For example, it is possible that computer-generated faces, such as those used in the PFPB, do not tap into face expertise as effectively as real images of faces^64^. As such, the current results for age judgements should be treated with some caution. A similar point applies to the conclusions from the gender task, which also used computer-generated stimuli. Given the recent surge of computer-generated face stimuli in research, it is important to understand for which tasks and in which populations conclusions drawn from computer-generated stimuli are generalisable to real-life performance; to date, though, there is very little work examining how gender and age judgements are affected by the use of computer-generated stimuli^65^.

In sum, our results indicate that a variety of face perception measures (identity-based and some that are non-identity-based) predict face recognition ability, and support the idea that more generalised face perception deficits underpin many cases of DP, in line with cognitive and neural models of face recognition (e.g.,^27,29^). However, the data reported here show little consistency in the type of perceptual judgements or tasks that predicted face memory, suggesting that at least some of these relationships may be driven by task-based factors (e.g., task demands, stimuli). These findings have implications for the inclusion of face perception tasks in diagnosis and further research in DP—first, they demonstrate the utility of including a broad array of tasks, including non-identity-tasks, when assessing face recognition abilities. Second, they highlight the need for research examining shared and unique mechanisms underpinning different face perception tasks. For example, understanding the potential mechanisms that are being tapped by the gender perception task and the CFMT and famous faces tasks may offer further insight into the nature of deficits in DP, and potential avenues for remediation.

### Supplementary Information

Supplementary Information.

## Data Availability

The data supporting this manuscript is available in OSF: https://osf.io/va4jh/.
