# Supplementary material for: Both identity and non-identity face perception tasks predict developmental prosopagnosia and face recognition ability
Source: Sci Rep. 2024 Mar 19;14:6626. doi: 10.1038/s41598-024-57176-x (PMC10951298; doi:10.1038/s41598-024-57176-x)
Supplement: Supplementary file 1 — Supplementary Information. [file 41598_2024_57176_MOESM1_ESM.pdf]

Supplementary materials for 'Both identity and non-identity face perception tasks predict developmental prosopagnosia and face recognition ability'

Rachel J Bennetts, Nicola J Gregory, & Sarah Bate

Data supporting these analyses is available on OSF

Table S1

Descriptive statistics for DP and control groups in each face processing task

Task		DP participants (N = 30)	Control participants (N = 75)
CFPT (% correct)			
	Mean	57.34	72.43
	SD	10.19	10.84
Face matching			
	Mean	1.53	1.99
	SD	0.35	0.52
RMITE			
	Mean	24.60	27.15
	SD	3.69	3.52
Ekman 60 faces			
	Mean	48.33	49.87
	SD	4.96	5.05
PFPB age			
	Mean	65.4	65.73
	SD	6.30	7.34
PFPB gender			
	Mean	57.83	62.85
	SD	9.65	8.29
Scrambled scenes d'			
	Mean	2.76	2.92
	SD	0.50	0.36
Two-tone face detection d'			
	Mean	2.39	2.95
	SD	0.88	0.59
Scrambled scenes RT			
	Mean	1738.53	1655.79
	SD	389.87	416.54
Two-tone face detection RT			
	Mean	2196.5	1706.78
	SD	605.65	484.32

Table S2
Reliability for DP and control groups in each face processing task

Task	DP participants		Control participants	
	Cronbach's α	Guttman's λ2	Cronbach's α	Guttman's λ2
CFPT (upright)	.663	.787	.666	.681
Face matching				
Same identity trials	.660	.695	.515	.558
Different identity trials	.739	.763	.557	.595
Ekman 60 faces	.719	.760	.695	.722
RMITE	.539	.612	.849	.855
PFPB				
Age	.928	.937	.890	.899
Gender	.915	.925	.903	.914
Scrambled scenes	.343	.453	.273	.349
Two-tone faces	.956	.959	.991	.991

Table S3
Coefficients for logistic regressions reported in text

Predictors	*b*	SE	beta	OR	OR CI (upper)	OR CI (lower)	*z*	Wald Statistic	*p*
Identity face perception tests only									
(Intercept)	1.90	0.84	1.38	6.70	1.30	34.60	2.27	5.15	0.023
Participant age	-0.01	0.02	-0.19	0.99	0.96	1.02	-0.71	0.50	0.480
CFPT	1.33	0.35	1.32	3.77	1.91	7.46	3.82	14.57	**< .001**
Face matching	0.69	0.32	0.66	2.00	1.06	3.77	2.13	4.54	**0.033**
Non-identity face perception tasks only									
(Intercept)	1.34	0.76	1.18	3.83	0.87	16.91	1.78	3.15	0.076
Participant age	-0.00	0.02	-0.06	1.00	0.96	1.03	-0.21	0.04	0.832
RMITE	0.49	0.28	0.50	1.64	0.94	2.85	1.75	3.05	0.081
Ekman	0.07	0.32	0.07	1.07	0.58	1.99	0.21	0.05	0.830
PFPB Age	-0.65	0.36	-0.65	0.52	0.26	1.07	-1.78	3.18	0.075
PFPB Gender	0.64	0.28	0.64	1.90	1.11	3.27	2.34	5.46	**0.019**
Scrambled scenes (*d'*)	0.23	0.27	0.23	1.26	0.74	2.15	0.87	0.75	0.387
Two-tone faces (*d'*)	0.80	0.30	0.80	2.23	1.24	4.02	2.67	7.15	**0.008**
Non-identity face perception tasks, controlling for identity tasks									
(Intercept)	0.68	0.98	1.88	1.97	0.29	13.50	0.69	0.48	0.490
Participant age	0.03	0.02	0.48	1.03	0.98	1.08	1.29	1.67	0.196
RMITE	0.67	0.40	0.67	1.94	0.88	4.28	1.65	2.72	0.099
Ekman	0.00	0.41	0.00	1.00	0.45	2.22	0.00	0.00	0.998
PFPB Age	-1.52	0.55	-1.52	0.22	0.07	0.64	-2.76	7.63	**0.006**
PFPB Gender	1.02	0.41	1.03	2.78	1.24	6.27	2.47	6.10	**0.014**
Scrambled scenes (*d'*)	0.18	0.37	0.17	1.19	0.58	2.46	0.47	0.22	0.637
Two-tone faces (*d'*)	0.97	0.39	0.98	2.64	1.24	5.65	2.51	6.29	**0.012**
CFPT	1.82	0.53	1.80	6.18	2.19	17.48	3.44	11.81	**< .001**
Face matching	1.03	0.45	0.98	2.79	1.16	6.75	2.28	5.21	**0.022**

Table S4
Coefficients for linear regressions reported in text with CFMT as outcome variable

Predictors	*b*	SE	95% CI (lower)	95% CI (upper)	beta	*t*	*p*
Identity face perception tests only							
(Intercept)	52.44	2.59	47.30	57.58		20.24	< .001
Participant age	-0.03	0.06	-0.14	0.08	-0.04	-0.50	0.619
CFPT	5.51	1.00	3.51	7.50	0.45	5.48	**< .001**
Face matching	4.47	1.04	2.41	6.53	0.35	4.30	**< .001**
Non-identity face perception tasks only							
(Intercept)	55.09	3.28	48.59	61.59		16.82	< .001
Participant age	-0.09	0.07	-0.23	0.06	-0.11	-1.20	0.234
RMITE	1.55	1.24	-0.91	4.00	0.13	1.25	0.214
Ekman	1.77	1.34	-0.90	4.44	0.15	1.32	0.190
PFPB Age	-0.51	1.41	-3.31	2.29	-0.04	-0.36	0.719
PFPB Gender	2.52	1.17	0.21	4.84	0.21	2.16	**0.033**
Scrambled scenes (*d'*)	1.08	1.12	-1.14	3.31	0.09	0.96	0.338
Two-tone faces (*d'*)	2.88	1.28	0.45	5.32	0.24	2.35	**0.021**
Non-identity face perception tasks, controlling for identity tasks							
(Intercept)	51.27	2.68	45.95	56.59		19.14	< .001
Participant age	0.00	0.06	-0.12	0.12	0.00	0.00	0.997
RMITE	0.93	1.00	-1.05	2.91	0.08	0.93	0.353
Ekman	0.66	1.10	-1.53	2.86	0.06	0.60	0.548
PFPB Age	-1.55	1.14	-3.82	0.71	-0.13	-1.36	0.177
PFPB Gender	1.94	0.94	0.07	3.81	0.16	2.06	**0.042**
Scrambled scenes (*d'*)	0.98	0.92	-0.85	2.81	0.08	1.07	0.289
Two-tone faces (*d'*)	1.63	1.00	-0.36	3.61	0.13	1.63	0.107
CFPT	4.77	1.02	2.76	6.79	0.39	4.70	**< .001**
Face matching	3.96	1.09	1.80	6.13	0.31	3.64	**< .001**

Table S5
Coefficients for linear regressions reported in text with famous face recognition as outcome variable

Predictors	*b*	SE	95% CI (lower)	95% CI (upper)	beta	*t*	*p*
Identity face perception tests only							
(Intercept)	86.45	5.30	75.95	96.96		16.33	< .001
Participant age	-0.15	0.12	-0.38	0.08	-0.11	-1.32	0.189
CFPT	8.04	2.05	3.98	12.11	0.38	3.93	**< .001**
Face matching	4.68	2.12	0.48	8.89	0.21	2.21	**0.030**
Non-identity face perception tasks only							
(Intercept)	86.62	5.77	75.17	98.06		15.02	< .001
Participant age	-0.15	0.13	-0.40	0.10	-0.11	-1.20	0.234
RMITE	5.91	2.20	1.54	10.27	0.28	2.68	**0.009**
Ekman	-0.68	2.37	-5.39	4.02	-0.03	-0.29	0.774
PFPB Age	-4.27	2.52	-9.27	0.73	-0.20	-1.69	0.094
PFPB Gender	5.20	2.09	1.06	9.35	0.24	2.49	**0.014**
Scrambled scenes (*d'*)	1.72	1.98	-2.22	5.65	0.08	0.87	0.389
Two-tone faces (*d'*)	4.19	2.16	-0.10	8.48	0.20	1.94	0.055
Non-identity face perception tasks, controlling for identity tasks							
(Intercept)	81.33	5.21	70.99	91.66		15.62	< .001
Participant age	-0.03	0.11	-0.26	0.20	-0.02	-0.27	0.787
RMITE	5.01	1.96	1.13	8.89	0.24	2.56	**0.012**
Ekman	-2.16	2.14	-6.41	2.10	-0.10	-1.01	0.317
PFPB Age	-5.75	2.25	-10.22	-1.28	-0.27	-2.56	**0.012**
PFPB Gender	4.45	1.85	0.77	8.13	0.21	2.40	**0.018**
Scrambled scenes (*d'*)	1.54	1.80	-2.03	5.11	0.07	0.86	0.395
Two-tone faces (*d'*)	2.45	1.94	-1.40	6.30	0.12	1.26	0.210
CFPT	6.83	1.98	2.90	10.76	0.32	3.45	**< .001**
Face matching	5.25	2.14	1.00	9.50	0.23	2.45	**0.016**

Table S6
Comparisons of regression coefficients for DP and control groups in linear regressions predicting CFMT scores.

**Coefficients**	**DP group B (SE B)**	**Control group B (SE B)**	***t***	***p***
Identity tasks only				
CFPT	**1.71 (0.96)**	**2.56 (1.07)**	0.59	.555
Face matching*	-1.31 (1.22)	**3.60 (0.95)**	3.17	.002
Non-identity tasks only				
PFPB Gender	-0.04 (0.92)	0.56 (1.10)	0.42	.677
Two tone faces	-1.32 (0.97)	1.36 (1.28)	1.67	.098
Non identity tasks, accounting for identity tasks				
PFPB Gender	0.21 (0.95)	0.52 (1.01)	0.22	.823

Note: $t(101)$ and p values from formulae in Cohen, Cohen, West, and Aiken[1], implemented by Soper[2]. Values in bold indicate that the coefficients were significant, $p < .05$. * indicates a significant difference between coefficients for the DP and control groups. Only variables that were significant in the overall regression analyses are presented.

Table S7
Comparisons of regression coefficients for DP and control groups in linear regressions predicting famous faces task scores.

Coefficients	DP group B (SE B)	Control group B (SE B)	*t*	*p*
Identity tasks only				
CFPT	1.01 (3.60)	-0.66 (1.11)	0.44	.658
Face matching	0.74 (4.58)	1.00 (0.97)	0.55	.955
Non-identity tasks only				
PFPB Gender	1.18 (3.05)	0.06 (1.07)	0.34	.730
RMITE	6.08 (3.30)	1.11 (1.07)	1.43	.155
Non identity tasks, accounting for identity tasks				
PFPB Gender	1.23 (3.26)	0.02 (1.07)	0.35	.725
PFPB Age	-4.33 (4.50)	0.17 (1.20)	0.97	.336
RMITE	6.24 (3.61)	1.14 (1.07)	1.96	.052

Note: $t(101)$ and p values from formulae in Cohen, Cohen, West, and Aiken[1], implemented by Soper[2]. Values in bold indicate that the coefficients were significant, $p < .05$. * indicates a significant difference between coefficients for the DP and control groups. Only variables that were significant in the overall regression analyses are presented.

Table S8
Coefficients for logistic regressions, including RT for face detection tasks.

Predictors	*b*	SE	beta	OR	OR CI (upper)	OR CI (lower)	*z*	Wald Statistic	*p*
Non-identity face perception tasks only									
(Intercept)	2.14	0.87	1.31	8.50	1.54	47.02	2.45	6.01	0.014
Participant age	-0.02	0.02	-0.31	0.98	0.95	1.02	-1.02	1.05	0.306
RMITE	0.54	0.30	0.54	1.72	0.96	3.07	1.82	3.30	0.069
Ekman	0.42	0.34	0.42	1.52	0.79	2.94	1.25	1.55	0.213
PFPB Age	-0.08	0.36	-0.09	0.92	0.45	1.87	-0.23	0.05	0.815
PFPB Gender	0.63	0.30	0.63	1.88	1.04	3.39	2.09	4.38	**0.036**
Scrambled scenes (RT)	0.65	0.37	0.65	1.91	0.93	3.93	1.77	3.13	0.077
Two-tone faces (RT)	-1.36	0.37	-1.35	0.26	0.12	0.53	-3.66	13.37	**< .001**
Non-identity face perception tasks, controlling for identity tasks									
(Intercept)	1.98	1.15	1.93	7.23	0.76	69.17	1.72	2.95	0.086
Participant age	0.00	0.03	0.02	1.00	0.95	1.05	0.05	2.19e-3	0.963
RMITE	0.68	0.42	0.68	1.97	0.87	4.45	1.62	2.64	0.104
Ekman	0.20	0.45	0.19	1.22	0.51	2.91	0.44	0.19	0.661
PFPB Age	-0.96	0.51	-0.96	0.38	0.14	1.05	-1.86	3.46	0.063
PFPB Gender	1.10	0.47	1.10	2.99	1.20	7.45	2.35	5.54	**0.019**
Scrambled scenes (RT)	0.99	0.53	1.00	2.70	0.95	7.68	1.86	3.48	0.062
Two-tone faces (RT)	-1.65	0.52	-1.67	0.19	0.07	0.54	-3.14	9.88	**0.002**
CFPT	1.86	0.54	1.83	6.41	2.24	18.33	3.47	12.02	< .001
Face matching	0.83	0.42	0.79	2.29	1.00	5.27	1.95	3.82	0.051

Table S9
Coefficients for linear regressions with CFMT as outcome variable, including RT for face detection tasks.

Predictors	*b*	SE	95% CI (lower)	95% CI (upper)	beta	*t*	*p*
Non-identity face perception tasks only							
(Intercept)	56.08	3.31	49.52	62.64		16.82	< .001
Participant age	-0.11	0.07	-0.25	0.03	-0.14	-1.51	0.13
RMITE	1.56	1.17	-0.76	3.88	0.13	1.33	0.19
Ekman	2.83	1.29	0.27	5.40	0.23	2.19	**0.03**
PFPB Age	1.53	1.36	-1.17	4.22	0.13	1.12	0.26
PFPB Gender	1.84	1.15	-0.45	4.12	0.15	1.59	0.11
Scrambled scenes RT	1.47	1.32	-1.16	4.09	0.12	1.11	0.27
Two-tone faces RT	-4.95	1.27	-7.46	-2.43	-0.40	-3.91	**< .001**
Non-identity face perception tasks, controlling for identity tasks							
(Intercept)	52.10	2.72	46.71	57.50		19.18	< .001
Participant age	-0.02	0.06	-0.14	0.10	-0.03	-0.33	0.742
RMITE	0.88	0.94	-1.00	2.75	0.07	0.93	0.356
Ekman	1.45	1.07	-0.68	3.57	0.12	1.35	0.180
PFPB Age	-0.13	1.11	-2.34	2.07	-0.01	-0.12	0.906
PFPB Gender	1.52	0.93	-0.32	3.36	0.12	1.64	0.104
Scrambled scenes RT	1.14	1.07	-0.98	3.26	0.09	1.07	0.287
Two-tone faces RT	-3.59	1.03	-5.64	-1.54	-0.29	-3.48	**< .001**
CFPT	4.76	0.96	2.85	6.67	0.39	4.95	**< .001**
Face matching	3.60	1.04	1.54	5.66	0.28	3.47	**< .001**

Table S10
Coefficients for linear regressions with famous face recognition as outcome variable, including RT for face detection tasks.

**Predictors**	***b***	**SE**	**95% CI (lower)**	**95% CI (upper)**	**beta**	***t***	***p***
Non-identity face perception tasks only							
(Intercept)	88.21	5.63	77.04	99.39		15.67	< .001
Participant age	-0.19	0.12	-0.44	0.05	-0.14	-1.55	0.125
RMITE	5.52	2.00	1.55	9.50	0.26	2.76	**0.007**
Ekman	1.20	2.20	-3.17	5.56	0.06	0.54	0.588
PFPB Age	-0.71	2.34	-5.34	3.93	-0.03	-0.30	0.762
PFPB Gender	3.86	1.99	-0.09	7.81	0.18	1.94	0.056
Scrambled scenes	3.24	2.24	-1.22	7.69	0.15	1.44	0.152
Two-tone faces	-9.79	2.15	-14.05	-5.52	-0.46	-4.55	**< .001**
Non-identity face perception tasks, controlling for identity tasks							
(Intercept)	81.33	5.21	70.99	91.66		15.62	< .001
Participant age	-0.03	0.11	-0.26	0.19	-0.02	-0.27	0.533
RMITE	5.01	1.96	1.13	8.89	0.24	2.56	**0.012**
Ekman	-2.16	2.14	-6.41	2.10	-0.10	-1.01	0.782
PFPB Age	-5.753	2.25	-10.22	-1.28	-0.27	-2.56	0.168
PFPB Gender	4.45	1.85	0.77	8.13	0.21	2.40	0.051
Scrambled scenes	1.55	1.80	-2.03	5.11	0.07	0.86	0.172
Two-tone faces	2.45	1.94	-1.40	6.30	0.12	1.26	**< .001**
CFPT	6.83	1.98	2.90	10.76	0.32	3.45	**< .001**
Face matching	5.25	2.14	1.00	9.50	0.23	2.45	**0.026**

Supplementary References

1. Cohen, J., Cohen, P., West, S. G. & Aiken, L. S. *Applied Multiple Regression/Correlation Analysis for the Behavioral Sciences*. (Routledge, Mahwah, 2022).
2. Soper, D. S. Significance of the Difference between Two Slopes Calculator [Software]. https://www.danielsoper.com/statcalc/calculator.aspx?id=103 (2023).
